# Professionalism and associated factors among health professions education students: A primary mixed-methods study from Qatar

**DOI:** 10.1371/journal.pone.0338913

**Published:** 2026-02-02

**Authors:** Dalal Hammoudi Halat, Banan Mukhalalati, Aaliah Aly, Mohammed Seed Ahmed, Alaa Daud, Ahmed Malki

**Affiliations:** 1 QH Health Office of Assessment and Accreditation, QU Health, Qatar University, Doha, Qatar; 2 QU Health Integrated Learning Office, QU Health, Qatar University, Doha, Qatar; 3 Clinical Pharmacy and Practice Department, College of Pharmacy, QU Health, Qatar University, Doha, Qatar; 4 Department of Basic Medical Sciences, College of Medicine, QU Health, Qatar University, Doha, Qatar; 5 Department of Pre-Clinical Oral Health Sciences, College of Dental Medicine, QU Health, Qatar University, Doha, Qatar; 6 Department of Biomedical Sciences, College of Health Sciences, QU Health, Qatar University, Doha, Qatar; Caleb University, NIGERIA

## Abstract

**Background:**

Professionalism, the judicious use of communication, knowledge, and values for benefits of individuals and communities, is a keystone in health education, and is demarcated by distinct tenets. Professionalism among health professions education students at Qatar University (QU) has not been previously explored. The current study aimed at assessment of professionalism and its tenets among students at the five QU Health Sector colleges, and exploring associated factors.

**Methods:**

A mixed-methods convergent parallel design was used. For the quantitative phase, data were collected anonymously from students using the Pharmacy Professionalism Instrument (PPI), which assesses overall professionalism score, and scores of six tenets: Excellence, Respect, Altruism, Duty, Accountability, and Honor/Integrity. The qualitative phase involved focus groups exploring students’ perspectives on professionalism and opportunities for its fostering.

**Results:**

A total of 536 survey responses were analyzed. The mean PPI score indicated fair levels of professionalism and Excellence, Altruism, Duty and Honor/Integrity tenets, while Accountability and Respect exhibited excellent levels. Scores statistically increased with study progress for overall professionalism, as well as Excellence and Respect tenets. Focus groups confirmed student views on tenets of Excellence, Respect, Altruism, Accountability, and Honor/Integrity. Yet, a slight discrepancy in the latter tenet emerged regarding necessity of reporting errors, with some students expressing concerns about potential consequences.

**Conclusion:**

Favorable professionalism development across years of study and some excellent professionalism tenets were revealed among students. Amidst evolution in education and sociocultural norms, health education curricula should uphold cultivation of professionalism concepts, and establish an environment of recognition and nurturing of professionalism.

## Introduction

The concept of professionalism has received increased attention in medical and health professions education over the past several years [1,2]. Traditionally, professionalism has been defined in terms of the traits associated with, or necessary for, the performance of a chosen “profession” [3]. In medicine, professionalism is defined as “the habitual and judicious use of communication, knowledge, technical skills, clinical reasoning, emotions, values, and reflection in daily practice, for the benefit of the individual and community being served” [4]. The basic characteristics of professionalism are deeply embedded in in morality and virtue [5]. However, its integration and standardization within health education and healthcare continuously undergo evolution, as a result of change in health academia and healthcare environments [6,7]. Across various health professions, definitions of professionalism vary from “active demonstration of the traits of a professional” to a detailed description of standards for that particular profession [8–10]. The importance of professionalism for health practitioners stems from being indispensable in the contract with society, a contract based on trust and placing the needs of patients above all other considerations [11].

Globally, regulatory and accrediting bodies have recommended professionalism as a component of both undergraduate and graduate health education curricula [12]. For example, the American Board of Internal Medicine (ABIM), the American College of Physicians Foundation, and the European Federation of Internal Medicine jointly authored a physician charter that established a set of professional responsibilities for how physicians should adhere to fundamental principles of patient welfare, patient autonomy, and justice [13]. Pharmacy education accrediting bodies require schools to foster and assess the development of student professionalism [14]. The Code of Ethics for Nurses requires them to consider for the welfare of individual patients, and to consider society as a whole and commit to the improvement of the quality of healthcare [15].

Although professionalism is considered a core competency that must be mastered during education and residency, it is rarely taught explicitly, but rather acquired through a “hidden” curriculum observed in various learning environments [16]. Such behaviors have been extensively addressed in literature [5,17,18] as well as by professional healthcare bodies. For instance, the ABIM defined six tenets of professionalism: *Altruism*, or prioritizing the best interest of patients above self-interest; *Accountability*, fulfilling obligations towards the profession, adhering to codes of conduct, and bearing responsibility towards patients, society, and the healthcare profession; *Excellence*, or producing quality work, commitment to exceed expectations both in responsibility towards patients and in life-long learning and knowledge acquisition; *Duty*; or appropriate care for patients regardless of circumstances; *Honor and Integrity*; being fair, truthful, straightforward, and keeping commitments; and *Respect* for others including patients and their families, as well as peers, thereby reflecting collegiality and teamwork [19]. Delineated by leaders in the medical field, this refined set of competencies has been included in health education and training, with emphasis on acceptable professional behavior and the effective and safe healthcare delivery [20–22]. Moreover, these core competencies were further refined to include additional attributes like servant leadership, emotional intelligence, self-awareness, and humility [23,24]. The incremental infusion of such attributes occurs in health education and training progressively, so that professionalism is not learnt at once, but rather is a skill that both, health education students and professionals, develop over a lifetime [25].

Several self-administered rating instruments have been developed to assess professionalism among health professions education students. For example, The Pennsylvania State College of Medicine (PSCOM) Professionalism Questionnaire is one of the first reliable surveys of professionalism attitudes among medical students, residents, and faculty, and was based on the ABIM tenets [26–28]. The Hisar’s instrument for nursing students proved a reliable tool as well [29], and was recommended according to best evidence synthesis results [30].

In pharmacy education, the Behavioral Professionalism Assessment Instrument [31], Lerkiatbundit’s professionalism scale [32], and the Professionalism Assessment Tool (PAT) [33], were reported. Another tool, the Pharmacy Professionalism Instrument (PPI), adapted the six tenets of the ABIM and proved to be a reliable measure of professionalism [34]. Subsequent studies demonstrated reliability of this instrument as well [35,36], and it was further used to assess development of professionalism through advancing classes across the curriculum [37]. Moreover, the PPI was used for medical students, as its tenets were described as highly applicable to these students and all healthcare education students alike [38]. In the Gulf region, a recent study used the PPI to assess professionalism among medical students from KSA, reflecting its utility in the social and cultural context of this region [39]. Nevertheless, many views consider professionalism to be multidimensional, requiring breakdown of several aspects, whereby the depth and quality of assessment go beyond the measurement of behavior to embrace diverse approaches for attainment of professional identity [40].

The Qatar University (QU) Health Sector includes 5 colleges: College of Medicine (CMED), College of Dental Medicine (CDEM), College of Nursing (CNURS), College of Pharmacy (CPH) and College of Health Sciences (CHS), with over 15 different graduate and undergraduate programs. Across the Health Sector, professionalism is a cornerstone of education, seamlessly integrated into academic programs. Starting from the first year, curricula across these programs emphasize ethical conduct, cultural sensitivity, and collaborative practice, ensuring students are equipped to meet the demands of the healthcare field. Through structured coursework, students explore professional values such as integrity, accountability, and patient-centered care, and these are refined later throughout experiential learning opportunities, further reinforcing professionalism by immersing students in real-world scenarios. Furthermore, interprofessional education initiatives foster collaboration among students from various health disciplines, promoting mutual respect and commitment to high-quality patient care, and ensuring graduates embody professionalism required to excel as compassionate healthcare providers in a globalized world [41,42].

Despite its importance, research on how health professions education students from different specialties collectively demonstrate professionalism, especially in diverse sociocultural and pedagogical contexts, remains scarce. Understanding how professionalism is cultivated across education can provide valuable insights for global health professions education, inform best practices, and address training gaps that affect healthcare delivery worldwide. Furthermore, no previous assessment has been realized to explore student-rated professionalism among health professions education students at the QU Health Sector nor in Qatar. The objectives of the current study were to assess professionalism and its tenets across students from different health professions educations programs and levels at QU Health Sector, and determine associated factors.

## Methods

### Study setting and design

This study was conducted across all five colleges at the QU Health sector: namely the College of Medicine (CMED), College of Dental Medicine (CDEM), College of Nursing (CNUR), College of Pharmacy (CPH), and College of Health Sciences (CHS). The study took place between January and November 2024.

A convergent parallel mixed-methods design was employed [43], whereby quantitative data were collected through a structured survey and qualitative data through focus group discussions. The two datasets were collected independently and subsequently integrated at the interpretation stage to ensure a comprehensive understanding of professionalism among QU Health students.

This design was selected because the survey component enabled data collection from a large number of participants across all health colleges, whereas the focus group allowed for an in-depth exploration of student’s perceptions of professionalism and its key tenets. Ethical approval for this study was obtained from the QU Institutional Review Board (QU-IRB 1977-EA/23). Participation in both study phases was entirely voluntary, and all participants were assured full anonymity, privacy, and data security. All study-related documents were securely stored on a password-protected laptop accessible only to the research team.

### Quantitative phase

#### Population and sampling.

A total population sampling approach was adopted to ensure representativeness across disciplines and academic levels. All undergraduate students enrolled in any of the five QU Health colleges, across all educational levels were invited to participate. This inclusive approach was selected to capture the full range of perspectives on professionalism across programs and educational stages, avoiding sampling bias and maximizing generalizability within the QU Health student population.

#### Data collection tool and procedure.

Data were collected using a self-administered structured survey assessing professionalism. The survey consisted of two sections: Section A: sociodemographic characteristics (gender, age, health college, and year of study), and Section B: the validated PPI tool developed by Chisholm et. al [34].

The PPI consists of 18 items scored on a five-point Likert scale (1 = strongly disagree to 5 = strongly agree), assessing the six tenets of professionalism— Excellence, Respect for Others, Altruism, Duty, Accountability, and Honor/Integrity—each represented by multiple items. The total professionalism score (maximum = 90) is obtained by summing all items, with separate subscale scores for each tenet: Excellence (5 items; maximum score: 25), Respect for others (4 items; maximum score = 20), Altruism (3 items; maximum score: 15), Duty (2 items; maximum score: 10), Accountability (2 items; maximum score: 10), and Honor/Integrity (2 items; maximum score: 10). Higher scores reflect higher levels of professionalism.

Due to the absence of established interpretation guidelines and to enable for a relative assessment of professionalism along with its subscales [44], scores were categorized using percentile thresholds: below the 10^th^ percentile as poor, between the 10^th^ and 50^th^ percentiles as fair, and above the 50^th^ percentile as excellent.

The full survey instrument was hosted on SurveyMonkey® (SurveyMonkey Inc., San Mateo, California, USA; 2024), secured with password protection. A link to the survey was generated to send to participants and engage them in the study. For collection of responses, an email with the survey link was sent to all undergraduate students enrolled in the QU health colleges through offices of the Assistant Deans for Student Affairs at each college. The email explained briefly the purpose of the study and reassured of anonymity, confidentiality, and voluntary nature of the participation with no associated benefits nor risks. Completion and submission of the survey were considered as informed consent. The survey remained open from January through September 2024, with reminders sent each 6 weeks to enhance participation.

#### Data analysis.

For analysis of survey results, quantitative descriptive and inferential analyses were conducted using SPSS software (IBM SPSS Statistics for Windows, version 27.0; IBM Corp., Armonk, NY, USA). Descriptive statistics, including frequencies (%), mean (± SD), and median (IQR), were used to summarize participants’ sociodemographic characteristics and responses to the PPI scale. For inferential analysis, parametric statistics were used to compare various subgroups regarding their overall perception of professionalism and their specific perceptions of each professionalism tenet/subscale. Independent *t*-tests were used for normally distributed variables with two groups, while one-way ANOVA was applied for comparisons involving more than two groups. When significant differences were detected, Bonferroni post-hoc tests were conducted to determine between-group differences. A significance level of p < 0.05 was established a priori. The internal consistency and reliability of the PPI total and subscale scores were evaluated using Cronbach’s alpha.

### Qualitative phase

#### Population and sampling.

A purposive sampling strategy was employed to ensure diversity and depth of perspectives. Participants were recruited from the same population as in the quantitative phase — all undergraduate students enrolled in any of the QU Health colleges were eligible to participate in the focus groups (FGs). Students were included if they expressed willingness to share their experiences and perceptions of professionalism.

To ensure representative participation, five FGs were conducted—one for each college. Each FG included approximately six students, with efforts made to achieve diversity across academic years and gender (where applicable; CHS is a female-only college). Recruitment was coordinated with class representatives, who disseminated invitations and study details. Interested students received an invitation email 1–2 weeks prior to the FG, including study information, confidentiality assurances, and consent procedures. They were also asked to sign a consent form on agreement to participate and to have their responses recorded for research purposes.

#### Data collection.

A semi-structured topic guide containing 10 open-ended questions, along with probes, was developed and conceptualized based on the research objectives and the PPI professionalism tenets. The aim of the focus groups was to explore students’ perspectives on the impact of professionalism, opportunities within QU Health Sector to foster professionalism, and their understanding of, as well as ways to demonstrate, the six professionalism tenets.

All FGs were conducted online using Microsoft Teams^®^ during May, June, and October 2024, considering availability and time preferences of the students. Each FG lasted about 45–50 minutes, and was conducted in English, recorded, transcribed verbatim and double-checked for accuracy. Once each FG started, no participants dropped out. One researcher moderated all the FGs to ensure consistency, and another researcher joined the FG to keep track of the discussions. At the introduction of the focus groups, participants were debriefed about the study objectives and voluntary participation. Also, the de-anonymity of their responses was emphasized through using alphanumerical codes for each participant, whereby these codes are only accessible to the research team and are password protected. After the fifth FG, a saturation point was reached as no new ideas emerged, and further data collection through FG was deemed unnecessary.

#### Data analysis.

The NVivo^®^ software (version 12 QSR International, Melbourne, Australia) was used to perform a thematic analysis combining both deductive and inductive approaches. The deductive thematic analysis was both iterative and interpretative [45] involving the organization of collected data into pre-determined categories based on a previous conceptual framework [12]. While frameworks on professionalism are relatively scarce, this particular framework was selected for several compelling reasons. It is a professionalism education framework developed through multidisciplinary consensus, making it highly relevant to this research, which includes all health education programs within the Health Sector at QU. Additionally, the framework is clear and comprehensive, incorporating the six tenets of professionalism along with other key professionalism attributes, all mapped across three core domains: individual proficiency, client-centeredness, and work-centeredness.

Concurrently, an inductive approach was used to explore the perceived impact of possessing professionalism and to identify opportunities within QU Health for fostering professionalism. The generated codes were then organized into themes and subthemes, and in instances of disagreement between researchers involved in the coding process, consensus was reached through discussion. Reflexivity was maintained by the researchers involved in the data analysis, being cognizant of their own personal context as educators and healthcare professionals, and of any potential effect this may exert on their interpretation of the data [46].

## Results

### Quantitative phase

The total student population at QU Health Sector at the time of the study consisted of 1,736 students, and the minimum required sample size for a 95% confidence interval was 315 and for a 99% confidence interval was 418. The response rate among QU Health Sector students on the survey was 34.6% (n = 600 out of 1,736), while the usable response rate was 89.3% (n = 536 out of 600), after excluding those who did not complete the survey, which is higher than the calculated minimum sample size. **Table 1** displays the demographic and professional characteristics of the participants, the majority of whom were females of age group 20–22 years. Furthermore, most participants were enrolled in the CMED and primarily in their second professional year.

**Table 1:** Demographic and professional characteristics of the health professions students (N = 536).

Characteristics	Frequency n [%]
**Gender**	**n = 536**
Female	409 [76.3]
Male	127 [23.7]
**Age (years)**	**n = 536**
**17-19**	214 [39.9]
**20-22**	282 [52.6]
**23-25**	40 [7.5]
**College**	**n = 536**
Medicine	282 [52.6]
Dental Medicine	89 [16.6]
Health Sciences	102 [19.0]
Pharmacy	45 [8.4]
Nursing	18 [3.4]
**HS Program**	**n = 102**
Biomedical Sciences	41 [40.2]
Public Health	15 [14.7]
Human Nutrition	26 [25.5]
Rehabilitation Sciences	20 [19.6]
**Current professional year** ^**a**^	**n = 536**
1	127 [23.7]
2	171 [31.9]
3	110 [20.5]
4	71 [13.3]
5	57 [10.6]

^a^Current professional year represents the academic year of studying a student’s chosen health profession.

**Table 2** presents responses for each individual item, organized according to the six tenets of professionalism: I. Excellence, II. Respect, III. Altruism, IV. Duty, V. Accountability, and VI. Honor/Integrity. **Table 3** details the possible and obtained scores, along with the scoring system for the PPI and each of its subscales/tenets. The overall mean score for the PPI was 75.9 (SD = 11.14), indicating a fair level of professionalism among students. The mean scores for the individual scale/tenets were as follows: Excellence, 21.5 (SD = 3.38); Altruism, 12.6 (SD = 2.20); Duty, 7.9 (SD = 1.80); and Honor/Integrity, 8.6 (SD = 1.50), all reflecting fair levels. In contrast, Accountability and Respect exhibited excellent levels, with mean scores of 8.3 (SD = 1.60) and 17.1 (SD = 2.80), respectively. Reliability analysis indicated a Cronbach’s alpha of 0.93 for the total PPI score, with subscale/tenet values of 0.83, 0.82, 0.70, 0.58, 0.66, and 0.58, respectively.

**Table 2:** Health professions students’ responses to pharmacy professionalism instrument (PPI) items evaluating the six tenets of professionalism.

Domain/Item	Strongly Disagree n (%)	Disagree n (%)	Neutral n (%)	Agree n (%)	Strongly Agree n (%)	Mean (±SD)	Median (IQR)	Missing n (%)
**Tenet I: Excellence**								
**I want to exceed the expectations of others.**	14 (2.6)	18 (3.4)	71 (13.2)	195 (36.4)	238 (44.4)	4.2 (0.96)	4 (1)	0 (0)
**It is important for me to produce quality work.**	15 (2.8)	2 (0.4)	17 (3.2)	134 (25.0)	368 (68.6)	4.6 (0.82)	5 (1)	0 (0)
**I complete my assigned responsibilities.**	14 (2.6)	7 (1.3)	32 (6.0)	212 (39.6)	271(50.5)	4.3 (0.86)	5 (1)	0 (0)
**I am committed to helping others.**	14 (2.6)	8 (1.5)	51 (9.5)	214 (39.9)	249 (46.5)	4.3 (0.89)	4 (1)	0 (0)
**I accept constructive criticism.**	13 (2.4)	9 (1.7)	74 (13.8)	241 (45.0)	199 (37.1)	4.1 (0.88)	4 (1)	0 (0)
**Tenet II: Respect**								
**I address others using appropriate titles.**	15 (2.8)	7 (1.3)	43 (8.0)	182 (34.0)	289 (53.9)	4.3 (0.90)	5 (1)	0 (0)
**I am diplomatic when expressing ideas and opinions.**	12 (2.2)	18 (3.4)	102 (19.0)	223 (41.6)	181 (33.8)	4.0 (0.93)	4 (1)	0 (0)
**I accept decisions of those in authority (instructors, supervisors, seniors, pharmacists).**	13 (2.4)	8 (1.5)	57 (10.6)	257 (48.0)	201 (37.5)	4.2 (0.86)	4 (1)	0 (0)
**I am respectful to individuals who have different backgrounds than mine.**	12 (2.2)	2 (0.4)	17 (3.2)	151 (28.2)	354 (66.0)	4.6 (0.78)	5 (1)	0 (0)
**Tenet III: Altruism**								
**I do not expect anything in return when I help someone.**	12 (2.2)	15 (2.8)	57 (10.6)	197 (36.8)	255 (47.6)	4.2 (0.91)	4 (1)	0 (0)
**I would take a job where I felt I was needed and could make a difference, even if I get paid less than other positions.**	21 (3.9)	37 (6.9)	149 (27.8)	186 (34.7)	143 (26.7)	3.7 (1.1)	4 (2)	0 (0)
**I treat all patients with the same respect, regardless of their social or economic status or ability to pay.**	13 (2.4)	5 (0.9)	16 (3.0)	119 (22.2)	383 (71.5)	4.6 (0.81)	5 (1)	0 (0)
**Tenet IV: Duty**								
**I attend class/training/work daily.**	18 (3.4)	12 (2.2)	59 (11.0)	179 (33.4)	268 (50.0)	4.2 (0.97)	4.5 (1)	0 (0)
**If I realize that I will be late to class or to work, I contact the appropriate individual at the earliest possible time to inform them**	24 (4.5)	69 (12.9)	120 (22.4)	165 (30.7)	158 (29.5)	3.68 (1.2)	4 (2)	0 (0)
**Tenet V: Accountability**								
**If I do not fulfill my responsibilities, I readily accept the consequences.**	15 (2.8)	13 (2.4)	56 (10.5)	239 (44.6)	213 (39.7)	4.2 (0.91)	4 (1)	0 (0)
**I complete my assignments/duties independently and without supervision.**	12 (2.2)	22 (4.1)	62 (11.6)	229 (42.7)	211 (39.4)	4.1 (0.93)	4 (1)	0 (0)
**Tenet VI: Honor/Integrity**								
**It is wrong to cheat to achieve higher rewards (for example, grades or money)**	15 (2.8)	7 (1.3)	26 (4.9)	92 (17.2)	396 (73.8)	4.6 (0.87)	5 (1)	0 (0)
**I would report an error even if no one else was aware of the mistake.**	10 (1.9)	17 (3.2)	100 (18.7)	226 (42.1)	183 (34.1)	4.0 (0.91)	4 (1)	0 (0)

**Table 3:** Possible scores and scoring system for the total pharmacy professionalism instrument (PPI) and its subscales.

Variable	Possible score (min-max)	Obtained score (min-max)	Mean (SD) score	Scoring system
**PPI Total**	0-90	18-90	75.9 (11.14)	Poor: < 67 Fair: 67–77 Excellent: > 77
**Tenet I: Excellence**	0-25	5- 25	21.5 (3.38)	Poor: < 18 Fair: 18–22 Excellent: > 22
**Tenet II: Respect**	0-20	4 −20	17.1 (2.80)	Poor: < 15 Fair: 15–17 Excellent: > 17
**Tenet III: Altruism**	0-15	3 −15	12.6 (2.20)	Poor: < 10 Fair: 10–13 Excellent: > 13
**Tenet IV: Duty**	0-10	2 −10	7.9 (1.80)	Poor: < 6 Fair: 6–8 Excellent: > 8
**Tenet V: Accountability**	0-10	2 −10	8.3 (1.60)	Poor: < 6 Fair: 6–8 Excellent: > 8
**Tenet VI: Honor/Integrity**	0-10	2 −10	8.6 (1.50)	Poor: < 7 Fair: 7–9 Excellent: > 9

Participants’ perceptions regarding their level of Excellence are detailed in Tenet I in **Table 2** [Mean = 4.4 (SD = 0.77), Minimum–Maximum = 1–5]. Over three-quarters of participants (80.8%, n = 433) expressed a desire to exceed others’ expectations. A strong majority identified producing high-quality work as personally important (93.7%, n = 383) and reported that they complete their assigned responsibilities (90.2%, n = 483). Additionally, 86.4% (n = 463) stated their commitment to helping others, and 82.1% (n = 440) indicated accepting constructive criticism, although 13.8% (n = 74) remained neutral on this aspect.

Participants perceptions regarding their level of Respect are detailed in Tenet II in **Table 2** [Mean = 4.3 (SD = 0.75), Minimum–Maximum = 1–5]. Notably, 87.9% (n = 471) of participants reported that they consistently address others using appropriate titles. Additionally, 41.6% (n = 223) indicated they are diplomatic when expressing their ideas, while 75.4% (n = 404) expressed acceptance of decisions made by authority figures. Furthermore, a significant majority, 94.2% (n = 505), demonstrated respect for individuals from diverse backgrounds.

Participants perceptions regarding their level of Altruism are detailed in Tenet III in **Table 2** [Mean = 4.3 (SD = 0.84), Minimum–Maximum = 1–5]. The majority of participants, 84.4% (n = 452), indicated that they do not expect anything in return when helping others. Additionally, 61.4% (n = 329) expressed a willingness to accept a job where they felt needed and could make a difference, even if offered lower pay, while 26.7% (n = 143) remained neutral on this statement. Notably, an impressive 93.7% (n = 502) reported that they treat all patients with equal respect, regardless of patients’ social or economic status or their ability to pay.

Participants perceptions regarding their level of duty are detailed in Tenet IV in **Table 2** [Mean = 4.0 (SD = 0.90), Minimum–Maximum = 1–5]. Most of participants (83.4%, n = 447) reported attending class, training, or work daily, reflecting strong commitment. Regarding communication about potential lateness, 60.3% (n = 323) agreed that they would contact the appropriate individual as soon as possible, while 17.4% (n = 93) disagreed.

Participants perceptions regarding their level of accountability are detailed in Tenet V in **Table 2** [Mean = 4.1 (SD = 0.79), Minimum–Maximum = 1–5]. A significant majority of participants, 84.3% (n = 452), indicated that they readily accept consequences of not fullfilling their responsibilities. Additionally, 82.1% (n = 440) reported that they complete their assignments and duties independently.

Participants perceptions regarding their level of honor are detailed in Tenet VI in **Table 2** [Mean = 4.3 (SD = 0.74), Minimum–Maximum = 1–5]. A substantial majority, 91.1% (n = 488), agreed that it is wrong to cheat for higher rewards. In addition, 76.3% (n = 409) of participants indicated that they would report an error, even if it went unnoticed by others.

**Table 4** demonstrates differences in professionalism and its six tenets among QU Health Sector students based on demographic and professional characteristics. For the overall level of professionalism, no significant differences were found among the subgroups, except for the current professional year, which showed a statistically significant difference (p = 0.04). The Bonferroni test indicated that, as students’ progress in their professional education journey, they tend to exhibit higher levels of professionalism; however, students in their fourth professional year acted as an outlier in this trend. Specifically, the mean (SD) scores for each professional year are as follows: year 1 = 75.3 (11.5), year 2 = 76.0 (12.0), year 3 = 77.1 (7.6), and year 4 = 72.9 (14.5). This pattern was also evident in the Tenets of Excellence and Respect, with significant p-values of 0.008 and 0.02, respectively.

**Table 4:** Difference in the levels of the six professionalism tenets according to sociodemographic characteristics.

Variable	n (%)	Mean (±SD)	P-value ^#^
**PPI Total**			
**Gender**			
Female	409 (76.3)	76.3 (11.1)	0.88
Male	127 (23.7)	75.8 (11.2)	
**Current professional year**			
1	127 (23.7)	75.3 (11.5)	0.04*
2	171 (31.9)	76.0 (12.0)	
3	110 (20.5)	77.1 (7.6)	
4	71 (13.3)	72.9 (14.5)	
5	57 (10.6)	78.6 (7.4)	
**College**			
Medicine	282 (52.6)	75.9 (11.2)	0.59
Dental Medicine	89 (16.6)	77.0 (9.3)	
Health Sciences	102 (19.0)	74.5 (13.1)	
Nursing	18 (3.4)	76.3 (12.3)	
Pharmacy	45 (8.4)	77.0 (8.8)	
**HS program**			
Biomedical Sciences	41(40.2)	74.1 (15.7)	0.50
Public Health	15 (14.7)	70.9 (10.6)	
Human Nutrition	26 (25.5)	74.9 (12.6)	
Rehabilitation Sciences	20(19.6)	77.7 (13.1)	
**Tenet I: Excellence**			
**Gender**			
Female	409 (76.3)	21.9 (3.38)	0.53
Male	127 (23.7)	21.9 (3.37)	
**Current professional year**			
1	127 (23.7)	21.3 (3.5)	0.008*
2	171 (31.9)	21.5 (3.6)	
3	110 (20.5)	21.8 (2.4)	
4	71 (13.3)	20.4 (4.3)	
5	57 (10.6)	22.5 (2.4)	
**College**			
Medicine	282 (52.6)	21.9 (2.8)	0.088
Dental Medicine	89 (16.6)	20.6 (4.1)	
Health Sciences	102 (19.0)	21.5 (3.4)	
Nursing	18 (3.4)	22.0 (3.2)	
Pharmacy	45 (8.4)	21.8 (2.5)	
**HS program**			
Biomedical Sciences	41(40.2)	20.8 (4.7)	0.53
Public Health	15 (14.7)	19.5 (3.1)	
Human Nutrition	26 (25.5)	20.6 (3.9)	
Rehabilitation Sciences	20(19.6)	21.6 (3.6)	
**Tenet II: Respect**			
**Gender**			
Female	409 (76.3)	17.1 (2.7)	0.45
Male	127 (23.7)	17.1 (2.8)	
**Current professional year**			
1	127 (23.7)	16.7 (2.9)	0.02*
2	171 (31.9)	17.0 (2.9)	
3	110 (20.5)	17.7 (2.0)	
4	71 (13.3)	16.6 (3.5)	
5	57 (10.6)	17.7 (2.8)	
**College**			
Medicine	282 (52.6)	17.1 (2.8)	0.78
Dental Medicine	89 (16.6)	17.3 (2.4)	
Health Sciences	102 (19.0)	16.8 (3.0)	
Nursing	18 (3.4)	17.0 (2.8)	
Pharmacy	45 (8.4)	17.1 (2.8)	
**HS program**			
Biomedical Sciences	41(40.2)	16.6 (3.7)	0.93
Public Health	15 (14.7)	16.5 (2.4)	
Human Nutrition	26 (25.5)	17.0 (3.2)	
Rehabilitation Sciences	20(19.6)	17.0 (1.8)	
**Tenet III: Altruism**			
**Gender**			
Female	409 (76.3)	12.6 (2.3)	0.72
Male	127 (23.7)	12.4 (2.2)	
**Current professional year**			
1	127 (23.7)	12.6 (2.3)	0.11
2	171 (31.9)	12.6 (2.3)	
3	110 (20.5)	12.6 (1.8)	
4	71 (13.3)	12.0 (2.5)	
5	57 (10.6)	13.0 (1.8)	
**College**			
Medicine	282 (52.6)	12.6 (2.2)	0.98
Dental Medicine	89 (16.6)	12.7 (1.9)	
Health Sciences	102 (19.0)	12.5 (2.6)	
Nursing	18 (3.4)	12.4 (2.5)	
Pharmacy	45 (8.4)	12.6 (1.6)	
**HS program**			
Biomedical Sciences	41(40.2)	12.4 (2.9)	0.24
Public Health	15 (14.7)	11.6 (2.6)	
Human Nutrition	26 (25.5)	12.4 (2.5)	
Rehabilitation Sciences	20(19.6)	13.4 (2.0)	
**Tenet IV: Duty**			
**Gender**			
Female	409 (76.3)	7.9 (1.9)	0.47
Male	127 (23.7)	7.9 (1.8)	
**Current professional year**			
1	127 (23.7)	8.0 (1.7)	0.13
2	171 (31.9)	7.9 (1.8)	
3	110 (20.5)	7.8 (1.7)	
4	71 (13.3)	7.6 (2.2)	
5	57 (10.6)	8.4 (1.4)	
**College**			
Medicine	282 (52.6)	7.8 (1.8)	0.69
Dental Medicine	89 (16.6)	8.0 (1.7)	
Health Sciences	102 (19.0)	8.0 (1.8)	
Nursing	18 (3.4)	8.4 (1.6)	
Pharmacy	45 (8.4)	8.0 (1.7)	
**HS program**			
Biomedical Sciences	41(40.2)	7.7 (2.0)	0.35
Public Health	15 (14.7)	7.7 (1.8)	
Human Nutrition	26 (25.5)	8.1 (1.7)	
Rehabilitation Sciences	20(19.6)	8.5 (1.2)	
**Tenet V: Accountability**			
**Gender**			
Female	409 (76.3)	8.3 (1.6)	0.31
Male	127 (23.7)	8.3 (1.7)	
**Current professional year**			
1	127 (23.7)	8.2 (1.6)	0.32
2	171 (31.9)	8.4 (1.6)	
3	110 (20.5)	8.4 (1.3)	
4	71 (13.3)	8.0 (2.0)	
5	57 (10.6)	8.4 (1.4)	
**College**			
Medicine	282 (52.6)	8.3 (1.6)	0.65
Dental Medicine	89 (16.6)	8.3 (1.5)	
Health Sciences	102 (19.0)	8.2 (1.7)	
Nursing	18 (3.4)	7.9 (1.7)	
Pharmacy	45 (8.4)	8.5 (1.3)	
**HS program**			
Biomedical Sciences	41(40.2)	8.3 (1.9)	0.52
Public Health	15 (14.7)	7.6 (1.5)	
Human Nutrition	26 (25.5)	8.2 (1.7)	
Rehabilitation Sciences	20(19.6)	8.4 (1.3)	
**Tenet VI: Honor/Integrity**			
**Gender**			
Female	409 (76.3)	8.6 (1.4)	0.68
Male	127 (23.7)	8.6 (1.5)	
**Current professional year**			
1	127 (23.7)	8.5 (1.7)	0.28
2	171 (31.9)	8.7 (1.5)	
3	110 (20.5)	8.8 (1.2)	
4	71 (13.3)	8.3 (1.6)	
5	57 (10.6)	8.7 (1.3)	
**College**			
Medicine	282 (52.6)	8.6 (1.5)	0.22
Dental Medicine	89 (16.6)	8.8 (1.4)	
Health Sciences	102 (19.0)	8.4 (1.6)	
Nursing	18 (3.4)	8.5 (1.9)	
Pharmacy	45 (8.4)	9.0 (1.2)	
**HS program**			
Biomedical Sciences	41(40.2)	8.3 (2.0)	0.39
Public Health	15 (14.7)	7.9 (1.3)	
Human Nutrition	26 (25.5)	8.7 (1.5)	
Rehabilitation Sciences	20(19.6)	8.8 (1.1)	

#P-value < 0.05 calculated using Independent-Sample T- test or One-Way Anova.

HS: Health Sciences.

## Qualitative phase

Thirty-two students from various QU Health colleges were interviewed: seven from CPH, six from CNURS, seven from CMED, four from CHS, and eight from CDEM. Three themes were deductively generated using the Sow et al. conceptual framework [12], while one theme emerged inductively through the analysis process. The generated themes and subthemes are presented in Table 5.

**Table 5:** Themes and subthemes generated from the FGs.

Theme	Sub-theme
**Inductive theme**	
Theme 1. Fostering professionalism in health education and practice	Sub-theme 1.1 Impacts of demonstrating professionalism Sub-theme 1.2 Opportunities for professionalism cultivation
**Deductive themes**	
Theme 2. Evaluating and enhancing individual proficiency	Sub-theme 2.1 Strategies for ensuring work excellence Sub-theme 2.2 Measures to overcome discipline-specific challenges
Theme 3. Demonstrating patient-centered care	Subtheme 3.1 Promoting respect for diverse backgrounds Subtheme 3.2 Enhancing care delivery through altruistic practices
Theme 4. Exhibiting work-centeredness	Subtheme 4.1 Demonstrating responsibility and accountability in professional settings Subtheme 4.2 Upholding ethical standards in clinical and educational environments

### Theme 1. Fostering professionalism in health education and practice

This theme focused on exploring students’ perceptions of the impact of demonstrating professionalism and identifying the opportunities available within QU Health programs to foster its development.

#### Impacts of demonstrating professionalism.

Participants identified several positive outcomes associated with demonstrating professionalism in their work and interactions. One significant benefit was the establishment of clear boundaries between colleagues, faculty members, clinical supervisors, and patients. In this regard, an interviewee said:

*“Professionalism plays an important role in setting clear boundaries between colleagues, faculty members, clinical supervisors, and patients. It helps in defining roles and expectations, which will then result in respectful and effective interactions at all levels.”* [Speaker 18 - CMED]

Another student elaborated:

*“… it helps prevent miscommunication and misunderstanding, which in turn ensure the delivery of high-quality care.”* [Speaker 22 - CHS]

Many students also highlighted how professionalism serves as a guiding principle for actions and decisions. In this context, a participant said:

*“Professionalism is important because it helps in forming the standards for how things should work, how conflicts should be managed, and how clinical practices should be carried out. It’s like setting guidelines that everyone must follow.”* [Speaker 8 - CNURS]

Trust-building was also frequently emphasized as a key outcome. A student mentioned:

*“When we demonstrate professionalism, others gain confidence in our abilities and intentions, which in turn strengthens trust and promotes collaboration.”* [Speaker 3 - CPH]

Additionally, professionalism was recognized for its protective role.

*“It ensures that everyone’s work and efforts are respected and safeguarded. Without professionalism, there’s a risk that someone’s contributions could be disregarded or stolen.”* [Speaker 2 - CPH]

Finally, professionalism was recognized for promoting a smooth workflow.

*“Professionalism removes potential obstacles that could disrupt productivity or hinder the smooth flow of work, helping in creating an environment where tasks are completed efficiently and effectively.”* [Speaker 29- CDEM]

#### Opportunities for professionalism cultivation.

Almost all students acknowledged the vital role faculty members play in promoting professionalism, particularly during the early stages of their education. A participant said:

*“Before our clinical rotations, our faculty members are the main role models for professionalism. They show us how to interact with others, handle challenges, and reflect the values of our profession.”* [Speaker 26-CDEM]

Participants also praised the integration of both general and college-specific courses that address professionalism throughout the curriculum. One student shared:

*“Since our foundation year, we have been introduced to a range of courses that incorporate professionalism from various perspectives, some are general and some specific to our profession, pharmacy. I believe this is the main way we learn about professionalism, as these skills don’t develop overnight; they are cumulative and build over time.”* [Speaker 6 - CPH]

Another student said:

*“We don’t have a specific course on professionalism, but it is integrated into all our nursing courses, helping us understand its importance in practice.”* [Speaker 12 - CNURS]

Moreover, students highlighted how opportunities for interdisciplinary collaboration and hands-on experience contribute to their professional development.

*“I think, Interprofessional Education activities help us develop professionalism by teaching us how to collaborate with other professions. Understanding their roles and how we complement each other improves our ability to work professionally as a team.”* [Speaker 21 - CHS]

*“In clinical settings, both from our preceptors and fellow nurses, we have gained valuable insights into professionalism. Observing and learning from their interactions has deepened our understanding of what it means to be a professional in healthcare.”* [Speaker 26 - CNURS]

### Theme 2. Evaluating and enhancing individual proficiency

The second theme explored strategies used by QU Health students to assess and improve work quality and prevent disciplinary actions, ensuring individual proficiency.

#### Strategies for ensuring work excellence.

Self-reflection was often highlighted by students as a key strategy for monitoring and enhancing the quality of their work. One student mentioned:

*“… Reflecting on past experiences allows us to evaluate what we did well and what went wrong, providing us with a clear path for improvement and growth.”* [Speaker 4 - CPH]

Many participants also actively sought feedback from faculty members, clinical supervisors, and patients to ensure the quality of their work and performance. In this regard, a student said:

*“Feedback from faculty and supervisors, with their years of experience and deep knowledge of the field, offers priceless perspective. They can assess whether the work was done excellently or just ‘good enough,’ helping us strive for better performance.”* [Speaker 30 - CDEM]

Another student added:

*“As a student nurse in the clinical setting, I measure my professionalism by the satisfaction of the patients I care for and the feedback from my supervisors. If they are happy with the care I provide and offer positive feedback, it reflects the quality of my work. If not, it highlights areas where I can improve.”* [Speaker 10- CNURS]

Finally, opinions were divided regarding the role of grades in assessing the quality of students’ work and their excellence. In this context, one student argued:

*“While grades are commonly used to assess performance, I personally believe they do not define a student’s excellence. Sometimes, academic pressure and exams can affect performance, but that doesn’t reflect their true abilities. Many students are incredibly smart and capable outside of those high-pressure scenarios.”* [Speaker 15 - CMED]

On the other hand, a student who disagreed said:

*“I believe we can always refer to our grades, even though many might disagree. In a high-pressure field like healthcare, the ability to perform under pressure, manage complex situations, and still meet high standards is essential. So, I think grades are one way to monitor and ensure the quality of our work.”* [Speaker 17-CMED]

#### Measures to overcome discipline-specific challenges.

Students identified acknowledging the problem as the first step toward addressing any discipline-related issue. As one student mentioned:

*“The first step to solving any problem is identifying and admitting it first, especially when it’s related to discipline. Once I’ve recognized that there is an issue, I’ll do whatever it takes through taking proactive steps to find a solution.”* [Speaker 18 - CMED]

Several key strategies for avoiding disciplinary issues were highlighted, including the use of calendars and reminders, along with effective planning and prioritization*.*

*“A great way to stay disciplined is by using calendars to schedule and set reminders for everything, making sure I’m always up to date with tasks, assessments, and responsibilities.”* [Speaker 13 – CNURS]

*“I guess discipline-related issues happen when students don’t have clear, pre-set goals and priorities. I believe that when we organize our thoughts and prioritize tasks based on our goals, we can avoid falling into issues that negatively affect our performance*.” [Speaker 5 - CPH]

Additionally, setting small, achievable goals was identified by students as an effective strategy. A student mentioned:

*“Challenging myself and setting small and achievable goals really helps in staying on track. When we break things down into manageable steps, it’s easier to stay disciplined and avoid falling behind.”* [Speaker 30 - CDEM]

Lastly, seeking support was recommended after students had tried to manage on their own but continued to struggle. In this regard, a student said:

*“Seeking support is crucial, especially after you’ve tried to manage things on your own. Whether it’s from mentors, peers, or family, asking for help can provide guidance in improving discipline and managing time more effectively.”* [Speaker 12 - CNURS]

### Theme 3. Demonstrating patient-centered care

The third theme explored the strategies employed by QU Health students to ensure the benefit and well-being of their patients.

#### Promoting respect for diverse backgrounds.

Language and literacy accommodation were highlighted by students as essential for maintaining respect among colleagues and patients. In this regard, a student said:

*“I usually use a language that the person understands, and if there’s a language barrier, we can always ask for help from a translator to ensure the message is communicated clearly.”* [Speaker 13 - CNURS]

Another student added:

*“If I have a patient with a lower literacy level or someone without a medical background, I use simpler language, speak slowly, give them time to ask questions, and encourage them to stop me if they need further clarification.”* [Speaker 25 - CDEM]

In addition, cultural openness and reciprocity were identified as key measures of showing respect. A student mentioned:

*“Be open to different cultures and accept the differences you see, especially in a place like Qatar, where people from all over the world come together. Listen, learn about their cultures, and share your own. Treat others how you’d like to be treated, and this will help everyone understand each other better.”* [Speaker 22 - CHS]

Students also emphasized the importance of respecting patients’ autonomy and confidentiality. An interviewee said:

*“Respect can be demonstrated by honoring the patient’s autonomy, supporting their choices, and maintaining their confidentiality.”* [Speaker 11- CNURS]

Maintaining appropriate non-verbal communication was also highlighted as key aspect of demonstrating respect.

*“We demonstrate respect by being mindful of our body language and tone. It’s important to ensure that both are appropriate not only for the person we’re speaking to but also for the situation at hand.”* [Speaker 27- CDEM]

Finally, during conflicts, active listening and effective communication were considered crucial for resolving issues while maintaining respect.

*“In cases of conflict, I would first listen to their perspective and ask them to explain how they expected us to handle the situation. Then, I would share our own perspective as well. It’s about listening and open communication to find common ground.”* [Speaker 4 - CPH]

#### Enhancing care delivery through altruistic practices.

There was consensus among students that altruism fosters a healthier working environment by promoting selfless giving, rather than transactional relationships, which can significantly impact patient outcomes. An interviewee stated:

*“I believe being altruistic creates a healthier working environment. When we focus on selfless giving instead of just mutual exchange, it makes a real difference in how we care for patients. It leads to better teamwork, more compassion, and ultimately improves patient outcomes because everyone is focused on doing what’s best for the patient, not just what’s in it for them.”* [Speaker 12 - CNURS]

Students identified altruistic practices as prioritizing balanced patient wellness and supporting colleagues to ensure the best outcomes for all patients.

*“I believe we should always put the patient first, considering not only their health needs but also their financial concerns and preferences. The patient should have a voice in their care, and we must prioritize them over our own benefit. However, the extent to which we sacrifice for the patient should always be balanced and proportionate.”* [Speaker 30 - CDEM]

*“Helping colleagues by sharing knowledge and offering assistance whenever possible is essential. By supporting each other, we can ensure the best outcomes for our patients.”* [Speaker 13- CNURS]

However, while students acknowledged the importance of altruism, they also emphasized the need for setting boundaries. In this regard, a student said:

*“Altruism is complex and requires mindfulness. It’s important to ensure you are both mentally and physically well before offering help, as being unwell can unintentionally impact the quality of care you provide.”* [Speaker 17 -CMED]

### Theme 4. Exhibiting work-centeredness

The final theme explored how QU Health students demonstrate responsibility, accountability, and personal integrity, all of which contribute to fulfilling tasks effectively and efficiently.

#### Demonstrating responsibility and accountability in professional settings.

Students identified various ways to demonstrate responsibility and accountability in professional settings. Key strategies included meeting deadlines and maintaining strong organizational skills. In this regard, a participant said:

*“I think I show responsibility by managing my time effectively, breaking tasks into manageable steps, prioritizing them according to deadlines, and tackling them in order.”* [Speaker 14 - CMED]

Eagerness for success and self-motivation to take initiatives were also emphasized by participants. A student mentioned:

*“Being self-motivated and confident in taking initiative is important. In nursing, for example, we are responsible for caring for our patients. When action is needed, we must think critically and take the necessary steps, without waiting for instructions from colleagues, consultants, or doctors.”* [Speaker 8- CNURS]

Engaging in continuous learning to fill knowledge gaps was identified as another crucial way to demonstrate responsibility.

*“Continuous learning is part of our responsibility. If we encounter a term or procedure, we are unfamiliar with, taking the initiative to learn about it and understand it is part of our professional duty.”* [Speaker 7 - CPH]

Following practice protocols and adhering to established guidelines were also highlighted.

*“As healthcare professionals, I believe we demonstrate responsibility by adhering to the protocols and guidelines within the clinical setting.”* [Speaker 23 - CHS]

In cases of unintentional mistakes, acknowledging and reporting them to the relevant authorities was seen as a crucial way to demonstrate accountability.

*“Accountability is often seen when acknowledge our mistakes, even if no one else has noticed them. We should admit it to our senior, because the issue may not lie with us, it could be that the system itself needs improvement. By acknowledging our mistakes, everyone has the opportunity to learn from them, and in some cases, it can lead to improvements in the system.”* [Speaker 10 - CNURS]

#### Upholding ethical standards in clinical and educational environments.

Participants had differing opinions on how students should handle cheating or unethical behavior. Some suggested confronting and advising the individual directly. In this regard, a student said:

*“Honestly, I would talk to my colleague and offer some advice, but I wouldn’t report them to the instructor. I think guiding them is a better option because I’m not sure how the college would handle it, and I don’t want to risk their future.”* [Speaker 8 - CNURS]

Others emphasized the importance of immediate reporting, especially when there is a risk of harm to team members or when patient care could be compromised. A student mentioned:

*“Maybe if a group member uses AI without permission, and it affects our submission, I would report it to the faculty member. I’d explain that we did the work, but the student used AI in this specific section. This way, we won’t lose marks because of her actions, as it directly impacts me.”* [Speaker 22 - CHS]

Another student added:

*“If I were to witness an action by a student or a doctor that could potentially endanger a patient’s safety, I would not hesitate to raise the concern to a superior. Patient safety is always the top priority, and it’s crucial to address any situation that could harm the patient, no matter the circumstances.”* [Speaker 13 - CNURS]

### Data integration

**Table 6** presents the integration of data from both the quantitative and qualitative phases. It demonstrates how the quantitative findings informed and enriched the qualitative analysis, as well as how the quantitative data influenced themes in the qualitative phase. The integration of the two data types revealed both areas of alignment and divergence in students’ views on the core tenets of professionalism. Across both data sources, there was consistent evidence that students are committed to demonstrating work excellence, respect, altruism, and accountability. However, a slight discrepancy emerged between the two phases, particularly regarding students’ approaches to maintaining ethical standards in clinical and educational settings. While the quantitative phase indicated that students would report errors even if no one else was aware, the qualitative data revealed conflicting opinions on whether reporting errors is always necessary, with some students expressing concerns about potential negative consequences of such reporting.

**Table 6:** Integration of qualitative and quantitative findings.

Quantitative phase			Qualitative phase		
Aligning results					
Domain	Mean (SD)	Item	Agreement% vs disagreement%	Sub-theme	Quote
**Tenet I: Excellence**	21.5 (3.38)	I complete my assigned responsibilities.	90.1% vs. 3.9%	Demonstrating responsibility and accountability in professional settings	*“I think I show responsibility by managing my time effectively, breaking tasks into manageable steps, prioritizing them according to deadlines, and tackling them in order.”* [Speaker 14 - CMED]
		It is important for me to produce quality work.	93.6% vs. 3.2%	Strategies for ensuring work excellence	*“It is really important to maintain good quality of work as a healthcare provider, so I think reflecting on past experiences allows us to evaluate what we did well and what went wrong, providing us with a clear path for improvement and growth.”* [Speaker 4 - CPH]
		I am committed to helping others.	86.4% vs. 4.1%	Enhancing care delivery through altruistic practices	*“Helping colleagues by sharing knowledge and offering assistance whenever possible is essential. By supporting each other, we can ensure the best outcomes for our patients.”* [Speaker 13 - CNURS]
		I accept constructive criticism.	82.1% vs. 4.1%	Strategies for ensuring work excellence	*“Feedback from faculty and supervisors, with their years of experience and deep knowledge of the field, offers priceless perspective. They can assess whether the work was done excellently or just ‘good enough,’ helping us strive for better performance.”* [Speaker 30 - CDEM]
**Tenet II: Respect**	17.1 (2.80)	I am diplomatic when expressing ideas and opinions.	75.4% vs. 5.6%	Promoting respect for diverse backgrounds	*“… I would first listen to their perspective and ask them to explain how they expected us to handle the situation. Then, I would share our own perspective as well. It’s about listening and open communication to find common ground.”* [Speaker 4 - CPH]
		I am respectful to individuals who have different backgrounds than mine.	94.2% vs. 2.6%	Promoting respect for diverse backgrounds	*“Be open to different cultures and accept the differences you see, especially in a place like Qatar, where people from all over the world come together. Listen, learn about their cultures, and share your own. Treat others how you’d like to be treated, and this will help everyone understand each other better.”* [Speaker 22 - CHS]
**Tenet III: Altruism**	12.6 (2.20)	I do not expect anything in return when I help someone.	84.4% vs. 5.0%	Enhancing care delivery through altruistic practices	*“I believe being altruistic creates a healthier working environment. When we focus on selfless giving instead of just mutual exchange, it makes a real difference in how we care for patients. It leads to better teamwork, more compassion, and ultimately improves patient outcomes because everyone is focused on doing what’s best for the patient, not just what’s in it for them.”* [Speaker 12 - CNURS]
		I treat all patients with the same respect, regardless of their social or economic status or ability to pay.	93.7% vs. 3.3%	Enhancing care delivery through altruistic practices	*“I believe we should always put the patient first, considering not only their health needs but also their financial concerns and preferences. The patient should have a voice in their care, and we must prioritize them over our own benefit. However, the extent to which we sacrifice for the patient should always be balanced and proportionate.”* [Speaker 30 - CDEM]
**Tenet V: Accountability**	8.3 (1.60)	If I do not fulfill my responsibilities, I readily accept the consequences.	84.3% vs. 5.2%	Demonstrating responsibility and accountability in professional settings	*“As a leader, I’m responsible for the team’s actions, whether it’s a mistake in planning or any other mistake. When mistakes happen, I take accountability because no one is perfect. What matters is how we respond to those mistakes. As a leader, it’s my responsibility to acknowledge them and work with the team to understand what went wrong and how we can improve going forward.”* [Speaker 2 - CPH]
		I complete my assignments/duties independently and without supervision.	82.1% vs. 6.3%	Demonstrating responsibility and accountability in professional settings	*“Being self-motivated and confident in taking initiative is important. In nursing, for example, we are responsible for caring for our patients. When action is needed, we must think critically and take the necessary steps, without waiting for instructions from colleagues, consultants, or doctors.”* [Speaker 8 - CNURS]
**Tenet VI: Honor/Integrity**	8.6 (1.50)	I would report an error even if no one else was aware of the mistake.	76.4% vs. 5.0%	Upholding ethical standards in clinical and educational environments	*“If I were to witness an action by a student or a doctor that could potentially endanger a patient’s safety, I would not hesitate to raise the concern to a superior. Patient safety is always the top priority, and it’s crucial to address any situation that could harm the patient, no matter the circumstances.”* [Speaker 13 - CNURS]
**Contradicting results**					
**Tenet VI: Honor/Integrity**	8.6 (1.50)	I would report an error even if no one else was aware of the mistake.	76.2% vs. 5.1%	Upholding ethical standards in clinical and educational environments	*“Honestly, I would talk to my colleague and offer some advice, but I wouldn’t report them to the instructor. I think guiding them is a better option because I’m not sure how the college would handle it, and I don’t want to risk their future.”* [Speaker 8 - CNURS]

## Discussion

Through a cross-sectional survey and in-depth focus groups, this study provided a snapshot of various ideas about professionalism from health education students’ viewpoint at QU Health Sector, reflecting their knowledge, attitudes, and behaviors as health education students and as future healthcare providers. The exploration of the multifaceted construct of professionalism is of ultimate importance in health professions teaching institutions, as it investigates the virtues, behaviors and attitudes expected of future healthcare professionals. This study adds to the accumulating body of evidence on the awareness of health professions education students about professionalism and its sophistication, and reflects robustness of the QU Health Sector educational curricula in nurturing professionalism, as evident from results of the different PPI items and the students’ quotes in focus groups.

Among the key findings of this study are the fair levels of self-perceived professionalism, with excellent Accountability and Respect tenets, exhibited by health professions education students. In a recent investigation among medical and nursing students, dedication and respect were ranked first and third in terms of student opinions of best behaviors associated with professionalism [47]. Respect emerged as a key facet of professionalism, aligning with its well-documented role in health-related ethics and patient-centered care [40,48–50]. According to results of the current study, not only students’ self-evaluation of respect showed to be excellent, but they also expressed how they demonstrate respect for patients from diverse backgrounds as part of patient-centered care. The responses of students regarding the Respect tenet conform with previous designations invoking respect as an integral aspect of health-related ethics, and promoting respect to have both a cognitive dimension, or believing that patients have value, as well as a behavioral one, which means acting in harmony with such belief [51]. As for Accountability tenet, which also received excellent self-assessment by our students in the PPI, it was demonstrated in students’ approaches towards meeting deadlines and maintaining organizational skills, as well as a personal responsibility for success, self-improvement, and ownership of their mistakes. These opinions on Accountability align with previous descriptions of professionalism from a more global perspective, such as that of the American Nursing Association’s Code of Ethics, which defines Accountability as being “answerable to oneself and others for one’s own actions”, whereby health professionals are responsible for their own practice, work environment, and patient safety [52]. Both Respect and Accountability relate to strong hierarchal societal norms in the Arab World and the Gulf region, and are important cultural values not only in health professions but also across wide cultural contexts of communication and interpersonal acquaintances [53].

Furthermore, overall professionalism scores in this study significantly increased across years, this “quantitative” increase being statistically important in tenets of Excellence and Respect. Such increase in professionalism scores with year of study is in line with data reported elsewhere, conforming to the idea that professionalism is grown and improved, not made [54]. Also, with curricula at QU Health Sector including experiential components with progress in study, professionalism is expected to develops as students advance in their programs, observing more encounters and contacting an increasing number of patients and mentors. This professionalism development over the years indicates that students inculcate and develop their professional identities in early phases of the curriculum, then further develop them as they become more involved in the community of healthcare practitioners [55]. Our data indicate statistically significant increase in scores of Excellence and Respect, while AlKhater and Colleagues reported significant increase in scores of Excellence and Altruism among medical Saudi students [39]. In another study, Excellence, Altruism, and Respect subscales drew higher agreement from year 6 than from year 3 medical students [56]. In contrast, this increase across years of study was not reported by Fahs and Colleagues [35] who piloted the PPI over pharmacy students but did not detect a significant increase from first to third professional years. Also, no significant increase in professionalism score using PPI was detected in another investigation on medical students over different points in time [38]. In our study, although fourth year students’ data appeared as an outlier to the raising trend of professionalism across years of study, this can be justified by their total count of 71 compared to a minimum of 110 in first, second and third years. However, with our data mirroring Saudi findings, it could be possibly inferred that, in regional Gulf contexts of health professions education, increased exposure to different encounters across study years provides a sustained means towards cumulative professional identity development. Our data are also aligned with global contexts indicating the longitudinal nature of professional development, whereby repeated engagement with professional authentic activities fosters a deeper adoption of professionalism [57].

Professionalism scores did not significantly vary with gender, college, or program, a finding that can be viewed positively in terms of equity and consistency, whereby professionalism is taught and assessed unwaveringly across the QU Health Sector. This could reflect the success of consistent curricular approaches that emphasize universal values and behaviors associated with professionalism, and that students explicitly and extensively described in focus groups. It could also reflect the shared institutional context or norms that can overshadow differences among students. The development of professionalism among our students is gender-neutral, and this may be affected by uniform institutional environments, equal expectations, as well as moral and ethical norms that convey equity among male and female students. This can contrast with some findings elsewhere, whereby gender bias is reported to have long-lasting effects of students’ professionalism development [58], and a deeper study of this contrast is interesting in terms of a broader understanding of professionalism. However, knowing that professionalism is a social construct and is culture-sensitive [59], and with our sample of surveyed students being relatively homogenous in terms of demographics, educational experiences, and cultural backgrounds, this may have resulted in similarities in professionalism scores across specialties and genders with no cultural challenges in understanding professionalism [60]. While this cannot be readily confirmed from our results, it has implications for further exploration of professionalism among health professions education students from different backgrounds. This would illustrate both its universal and culture-specific aspects, thereby guiding into more informed incorporation of multi-cultural aspects of professionalism education in health curricula.

The overarching framework of professionalism identified through qualitative analysis was based on the three main themes of individual proficiency, patient-centered care, and work centeredness. Again, and by looking into the subthemes, Excellence, Respect and Accountability were reinforced tenets in the focus group discussions, with altruistic practices also emerging as a noticeable subtheme, alongside upholding ethical standards. Students demonstrated a commitment to excel in their work and to assess and enhance individual capacities, by strategies focused around self-reflection, planning and prioritization, setting achievable goals and seeking support. Such student views coincide with previous views of excellence as an intrinsic value of personal integrity and fulfillment, that must come from within the self as a matter of purpose and character, whereby individuals realize their full potential through work, and use excellence to attract more excellence [61]. In demonstrating Respect, students mentioned language, non-verbal communication, and active listening. This aligns with previous evidence showing that poor communication in healthcare might lead to adverse consequences, while effective communication is associated with positive outcomes including proper information flow, effective interventions, increased patient safety and employee morale, and more satisfaction of patients and their families [62]. Among strategies to demonstrate Accountability, students mentioned time management, continuous learning, self-motivation, and acknowledging mistakes. These attributes are so diverse to indicate awareness of students to the changing landscape of healthcare, thereby the need for self-improvement, as well as maintenance of standards when unintentional errors occur, coinciding with previous scholar opinions on professionalism [63,64]. Moreover, accountability among our students is an attribute that can be connected to wider global mandates in health education, whereby students are fostered to direct their education, service, and research towards addressing the priority health concerns of the community, region, or country they serve [65]. Notably, students’ views on altruistic practices expressed not only prioritizing patient needs and supporting colleagues, but also a forward-thinking perspective on the need to set boundaries while being altruistic. This distinction between personal and working life and being mindful of one’s own well-being before being able to support others has been mentioned in professionalism assays. The implications of such views from students on professionalism are noteworthy, in light of research indicating that over-commitment, work-life imbalance, poor physical health, and cognitive decline can be drivers of unprofessional behavior [66].

Apart from the aforementioned framework, the inductive theme on fostering professionalism and opportunities for its growth at the QU Health Sector engulfed many aspects like building trust and the importance of role models as well as interdisciplinary collaboration in development of the professional identity. The effect of strong positive role modeling in health education learning environments has been previously accentuated across professionalism studies in various contexts [67,68], including a related finding from the Gulf region, whereby medical students from Bahrain identified role models as essential to the development of professionalism [69]. Moreover, the role of interprofessional education in creating a professionalism framework that includes values shared by all health care professionals has been described previously in medical education literature [70], yet emphasized again with recent changes in healthcare workforce and prominence of successful teams and strong relationships [71]. With Qatar University spearheading efforts in the region in terms of interprofessional education, including working towards establishing it across curricula and facilitating collaborative practice [72], opportunities exist to explore how interprofessional activities shape behaviors of students and assists in their professionalism development through interaction among different professions, which could inform the decision making process in curricular innovation and update.

Upon comparison of the quantitative and qualitative data, some divergence appeared with respect to reporting errors. Despite that the student survey showed fair levels for Honor/Integrity, and that students would report errors even with no one watching, the focus groups revealed some discrepancies. Students may not report a classmate violating integrity standards in an exam or assignment to the instructor unless it directly affects them, but would report a major error that can put patient safety at stake. Care about patient safety and ultimate best outcomes for patients is a core professional value, and patient safety correlates significantly with professionalism [73]. Moreover, the failure to disclose a medical error, at least if a patient suffers major impairment, is not in accord with healthcare professional obligations [74]. The implications of such different views for students on reporting errors should be carefully scrutinized; while students should be encouraged to strongly uphold ethics when it comes to patients and health outcomes, they should be also effectively coached about remediation practices across health education curricula [75]. It should be reiterated to students that addressing professionalism early in their education is critical, and practicing it by a future healthcare professional is essential, regardless of the year of study, level of training, or nature of an observed error.

Our study has several strengths. We used a validated instrument that proved to be reliable according to previous literature [34,35], and we collected student-ratings from 5 colleges and different years of study, to have a representative student population. The majority of our surveyed students were females, in line with demographics at the QU Health Sector and the university in general. We also gauged the power of focus groups with different college, level, and gender representation, to gain deeper insights from students and taper on the cultural and human aspects that would subtly settle if addressed during discussions. This gave our findings the ability to effectively probe different perspectives on professionalism. Our instrument was brief and short, increasing the likelihood of students completing it without obvious survey fatigue, and avoiding participant drop off that is more likely with longer surveys. However, this study does have limitations. The survey and focus groups were done during a given unit of time, featuring a cross-sectional view, which, though feasible, does not allow purposeful follow-up of specific cohorts of students to see how their professionalism constructs develop across years of study and various exposures to faculty, mentors, academic competencies, applied skills, and peers. As such, longitudinal studies would be better suited to monitor development of professionalism and its tenets across health professions education students. Furthermore, the PPI was self-administered, raising the probability of bias and lack of attention to details by participating students. Despite that PPI does not relate to pharmacy in specific and has been used for other student groups, future studies should give priority to systematically advancing the performance of existing instruments for various health professions education students, and improving the assessment of tenets that exhibited the lowest Cronbach’s alpha values such as Duty and Honor/Integrity.

## Conclusion

This study is the first in Qatar investigating professionalism and its tenets among health professions education students through a mixed-methods approach. The findings highlighted fair levels of self-perceived professionalism, with excellence in Accountability and Respect tenets. As students progressed through their education years, notable improvements in professionalism scores were observed, reaffirming the notion that professionalism develops over time through structured curricula and varied learning experiences. However, the discrepancy in ethical decision-making, particularly regarding the necessity of error reporting, underscores some nuanced challenges in cultivating comprehensive professional attitudes.

Our results highlighted the critical role of various factors like institutional environment, role modeling, self-awareness and interdisciplinary collaboration, in fostering professionalism. While the absence of significant variation across demographic and academic variables indicates probable equity in professionalism education, it also reflects a need to further explore the influence of diverse cultural and personal experiences. The baseline findings reported here can pave the way for more advanced benchmarking of professionalism that can be led by QU Health Sector, through standardized curricular integration of professionalism, research on longitudinal professional development, exploring other national and regional norms, and training on cultural sensitivity and ethical practices.

## References

[1] Smith KJ, Farland MZ, Edwards M, Buring S, Childs GS, Dunleavy K, et al. Assessing professionalism in health profession degree programs: A scoping review. Curr Pharm Teach Learn. 2021;13(8):1078–98. doi: 10.1016/j.cptl.2021.06.006 34294251

[2] Sadeq A, Guraya SS, Fahey B, Clarke E, Bensaaud A, Doyle F, et al. Medical professionalism education: a systematic review of interventions, outcomes, and sustainability. Frontiers in Medicine. 2025;12. 10.3389/fmed.2025.1522411 PMC11911526 40098925

[3] Henry MC, Joseph K-A, Reyna C, Raja S, Stein SL. Redefining professionalism. Am J Surg. 2021;222(5):899–900. doi: 10.1016/j.amjsurg.2021.04.013 34001334

[4] Koornhof L. Professionalism is vital. South African Journal of Clinical Nutrition. 2025;38(3). doi: 10.1080/16070658.2025.2555658

[5] Bhardwaj A. Medical Professionalism in the Provision of Clinical Care in Healthcare Organizations. J Healthc Leadersh. 2022;14:183–9. doi: 10.2147/JHL.S383069 36320452 PMC9618247

[6] Ploylearmsang C. Health professionalism and health profession education in the 21st century: an example of pharmacy education. MedEdPublish. 2022;11:3. doi: 10.12688/mep.17425.2

[7] Marcotte LM, Moriates C, Wolfson DB, Frankel RM. Professionalism as the Bedrock of High-Value Care. Acad Med. 2020;95(6):864–7. doi: 10.1097/ACM.0000000000002858 31274519

[8] Unger J-P, Morales I, De Paepe P, Roland M. The physician and professionalism today: challenges to and strategies for ethical professional medical practice. BMC Health Serv Res. 2020;20(Suppl 2):1069. doi: 10.1186/s12913-020-05884-1 33292178 PMC7723462

[9] Parastesh S, Hosseini M, Mohammadi-Shahbolaghi F, Maddah SSB, Ebadi A. Exploring the new characteristics of professionalism in nursing from the perspective of experts: A qualitative Study. J Taibah Univ Med Sci. 2025;20(5):673–86. doi: 10.1016/j.jtumed.2025.09.003 41126846 PMC12538062

[10] Nasseripour M, Agouropoulos A, Van Harten MT, Correia M, Sabri N, Rollman A. Current State of Professionalism Curriculum in Oral Health Education. Eur J Dent Educ. 2025;29(1):92–103. doi: 10.1111/eje.13048 39501916 PMC11730743

[11] DeBenedectis CM. Professionalism Training in the Post-COVID-19 Era. J Am Coll Radiol. 2023;20(11):1146–51. doi: 10.1016/j.jacr.2023.04.004 37201690 PMC10186847

[12] Sow CF, Chandratilake M, Nadarajah VD. Developing a Professionalism Education Framework at the Institutional Level with Multidisciplinary Consensus. EIMJ. 2021;13(2):25–40. doi: 10.21315/eimj2021.13.2.3

[13] Chassin MR, Baker DW. Aiming higher to enhance professionalism: beyond accreditation and certification. JAMA. 2015;313(18):1795–6. doi: 10.1001/jama.2015.3818 25965212

[14] Murry LT, Murry JS, Pick A, Witry MJ. A Qualitative Exploration of ACPE Standard 4 Key Elements From the Perspective of Student Pharmacists. Am J Pharm Educ. 2023;87(12):100581. doi: 10.1016/j.ajpe.2023.100581 37517524

[15] Cao H, Song Y, Wu Y, Du Y, He X, Chen Y, et al. What is nursing professionalism? a concept analysis. BMC Nurs. 2023;22(1):34. doi: 10.1186/s12912-022-01161-0 36747180 PMC9902819

[16] Poola VP, Suh B, Parr T, Boehler M, Han H, Mellinger J. Medical students’ reflections on surgical educators’ professionalism: Contextual nuances in the hidden curriculum. Am J Surg. 2021;221(2):270–6. doi: 10.1016/j.amjsurg.2020.09.003 32943180

[17] Shelton W, Silberstein S, Campo-Engelstein L, Pohl H, Desemone J, Jacoby LH. Educational opportunities about ethics and professionalism in the clinical environment: surveys of 3rd year medical students to understand and address elements of the hidden curriculum. International Journal of Ethics Education. 2023;8(2):351–72. doi: 10.1007/s40889-023-00163-z

[18] Guraya SS, Kearney GP, Doyle F, Sadeq A, Bensaaud A, Clarke E, et al. “Busting the hidden curriculum” a realist and innovative perspective to foster professional behaviors. Front Med (Lausanne). 2024;11:1484058. doi: 10.3389/fmed.2024.1484058 39697199 PMC11652187

[19] Committees on Evaluation of Clinical Competence and Clinical Competence and Communication Programs AB. Project professionalism. Philadelphia, Penn: American Board of Internal Medicine. 2001.

[20] Bakunda L, Crooks R, Johnson N, Osei-Tutu K, Bharwani A, Gye E, et al. Redefining professionalism to improve health equity in competency based medical education (CBME): A qualitative study. MedEdPublish (2016). 2024;14:237. doi: 10.12688/mep.20489.1 39600517 PMC11589420

[21] Zeeman JM, Kiser SN, Steeb DR, Hubal R. Identifying Priority Student Leadership and Professionalism Attributes Among Faculty, Preceptors, and Students via Modified Delphi. Am J Pharm Educ. 2020;84(11):8076. doi: 10.5688/ajpe8076 34283754 PMC7712725

[22] Murphy S, Whitehouse L, Parsa B. Teaching professionalism: some features in Canadian physiotherapy programs. Physiotherapy Theory and Practice. 2020;36(5):615–27. 29958035 10.1080/09593985.2018.1491080

[23] Matchett CL, Usher EL, Ratelle JT, Suarez DA, Leep Hunderfund AN, Aragon Sierra AM, et al. Physician Humility: A Review and Call to Revive Virtue in Medicine. Ann Intern Med. 2024;177(9):1251–8. doi: 10.7326/M24-0842 39074373

[24] Partido BB, Stefanik D, Rashid W. Relationship between emotional intelligence and professionalism among second-year dental students. J Dent Educ. 2021;85(3):411–7. doi: 10.1002/jdd.12467 33124069

[25] Ludwig S. Professionalism. Pediatrics in Review. 2020;41(5):217–23. 32358027 10.1542/pir.2018-0234

[26] Blackall GF, Melnick SA, Shoop GH, George J, Lerner SM, Wilson PK, et al. Professionalism in medical education: the development and validation of a survey instrument to assess attitudes toward professionalism. Med Teach. 2007;29(2–3):e58-62. doi: 10.1080/01421590601044984 17701611

[27] Bustamante E, Sanabria Á. Spanish adaptation of The Penn State College of Medicine Scale to assess professionalism in medical students. Biomedica. 2014;34(2):291–9. doi: 10.1590/S0120-41572014000200015 24967934

[28] Tanrıverdi EÇ, Nas MA, Kaşali K, Layık ME, Abd El-Aty AM. Validity and reliability of the Professionalism Assessment Scale in Turkish medical students. PLoS One. 2023;18(1):e0281000. doi: 10.1371/journal.pone.0281000 36701346 PMC9879428

[29] Hisar F, Karadağ A, Kan A. Development of an instrument to measure professional attitudes in nursing students in Turkey. Nurse Educ Today. 2010;30(8):726–30. doi: 10.1016/j.nedt.2010.01.013 20378213

[30] Li H, Ding N, Zhang Y, Liu Y, Wen D. Assessing medical professionalism: A systematic review of instruments and their measurement properties. PLoS One. 2017;12(5):e0177321. doi: 10.1371/journal.pone.0177321 28498838 PMC5428933

[31] Hammer DP, Mason HL, Chalmers RK, Popovich NG, Rupp MT. Development and testing of an instrument to assess behavioral professionalism of pharmacy students. American Journal of Pharmaceutical Education. 2000;64(2):141–51.

[32] Lerkiatbundit S. Factor Structure and Cross-Validation of a Professionalism Scale in Pharmacy Students. Journal of Pharmacy Teaching. 2005;12(2):25–49. doi: 10.1300/j060v12n02_03

[33] Kelley KA, Stanke LD, Rabi SM, Kuba SE, Janke KK. Cross-validation of an instrument for measuring professionalism behaviors. Am J Pharm Educ. 2011;75(9):179. doi: 10.5688/ajpe759179 22171107 PMC3230340

[34] Chisholm MA, Cobb H, Duke L, McDuffie C, Kennedy WK. Am J Pharm Educ. 2006;70(4):85. 17136204 10.5688/aj700485 PMC1636969

[35] Fahs I, Nasser S, Bou Malhab S, Younes S, Safwan J, Bouraad E, et al. Assessment of professionalism among pharmacy students: A pilot study exploring professionalism tenets and associated factors. Pharm Educ. 2023;23(1):283–95. doi: 10.46542/pe.2023.231.283295

[36] Araújo-Neto F de C, Prado FO, Dosea AS, da Fonseca FL, de Araújo DCSA, Brito G de C, et al. Assessment of Professionalism in Pharmacists and Pharmacy Students: Scoping Review of Instruments and Validity Evidence. Am J Pharm Educ. 2024;88(8):100733. doi: 10.1016/j.ajpe.2024.100733 38866371

[37] Huang Y-M, Chan H-Y, Lee P-I, Tang Y-W, Chiou T-W, Liu KCSC, et al. Exploration of changes in pharmacy students’ perceptions of and attitudes towards professionalism: outcome of a community pharmacy experiential learning programme in Taiwan. BMC Med Educ. 2022;22(1):195. doi: 10.1186/s12909-022-03261-6 35313880 PMC8938161

[38] Nakamura M, Altshuler D, Chadwell M, Binienda J. Clinical skills development in student-run free clinic volunteers: a multi-trait, multi-measure study. BMC Med Educ. 2014;14:250. doi: 10.1186/s12909-014-0250-9 25495286 PMC4267714

[39] AlKhater SA. Perception of Saudi Undergraduate Students Towards Professionalism in Medicine. Sultan Qaboos Univ Med J. 2021;21(3):378–85. doi: 10.18295/squmj.4.2021.019 34522402 PMC8407911

[40] Fong W, Kwan YH, Yoon S, Phang JK, Thumboo J, Leung YY, et al. Assessment of medical professionalism: preliminary results of a qualitative study. BMC Med Educ. 2020;20(1):27. doi: 10.1186/s12909-020-1943-x 32000755 PMC6993492

[41] Al-Jayyousi GF, Abdul Rahim H, Alsayed Hassan D, Awada SM. Following Interprofessional Education: Health Education Students’ Experience in a Primary Interprofessional Care Setting. J Multidiscip Healthc. 2021;14:3253–65. doi: 10.2147/JMDH.S318110 34853515 PMC8628122

[42] Kane T, Chivese T, Al-Moslih A, Al-Mutawa NAM, Daher-Nashif S, Hashemi N, et al. A program evaluation reporting student perceptions of early clinical exposure to primary care at a new medical college in Qatar. BMC Med Educ. 2021;21(1):162. doi: 10.1186/s12909-021-02597-9 33731085 PMC7968227

[43] Almeida F. Strategies to perform a mixed methods study. European Journal of Education Studies. 2018.

[44] de Beurs E, Boehnke JR, Fried EI. Common measures or common metrics? A plea to harmonize measurement results. Clin Psychol Psychother. 2022;29(5):1755–67. doi: 10.1002/cpp.2742 35421265 PMC9796399

[45] Braun V, Clarke V. Using thematic analysis in psychology. Qual Res Psychol. 2006;3(2):77–101.

[46] Olmos-Vega FM, Stalmeijer RE, Varpio L, Kahlke R. A practical guide to reflexivity in qualitative research: AMEE Guide No. 149. Medical Teacher. 2023;45(3):241–51. 10.1080/0142159X.2022.2057287 35389310

[47] Ramanathan V, Pushpanathan P, Bodhare T, Bele S. Perceptions on Medical Professionalism Among Future Healthcare Professionals: A Mixed Method Study. Cureus. 2024;16(4):e57573. doi: 10.7759/cureus.57573 38707144 PMC11069035

[48] Chervenak FA, Makatsariya A, Degtyareva M, Savelyeva G, Bitsadze V, Strizhakov A, et al. Respect for professors: an often underappreciated component of professionalism in medical education. J Perinat Med. 2017;45(9):1079–80. doi: 10.1515/jpm-2017-0347 29182514

[49] Guraya SY, Sulaiman N, Guraya SS, Yusoff MSB, Roslan NS, Al Fahim M, et al. Understanding the climate of medical professionalism among university students; A multi-center study. Innovations in Education and Teaching International. 2020;58(3):351–60. doi: 10.1080/14703297.2020.1751237

[50] Sattar K, Yusoff MSB, Arifin WN, Yasin MAM, Nor MZM. Scoping Review of frequently highlighted attributes of Medical Professionalism in an Undergraduate Medical Education Context. Pak J Med Sci. 2021;37(4):1221–9. doi: 10.12669/pjms.37.4.4004 34290812 PMC8281152

[51] Beach MC, Duggan PS, Cassel CK, Geller G. What does “respect” mean? Exploring the moral obligation of health professionals to respect patients. J Gen Intern Med. 2007;22(5):692–5. doi: 10.1007/s11606-006-0054-7 17443381 PMC1852905

[52] Enwereuzo PN, Amuma NS. Institutional leadership and professional practices in tertiary institutions in Rivers State. Journal of Education and Society. 2022;12(2):2657–75.

[53] Khosh NK, Khalil AA, Alhaded HHS. Cultural values and norms of communication: A view from the Middle East. In: Proceedings of ADVED, 2020.

[54] McWilliams JM. Professionalism Revealed: Rethinking Quality Improvement in the Wake of a Pandemic. NEJM Catalyst. 2020;1(5). doi: 10.1056/cat.20.0226

[55] Sattar K, Akram A, Ahmad T, Bashir U. Professionalism development of undergraduate medical students: Effect of time and transition. Medicine (Baltimore). 2021;100(9):e23580. doi: 10.1097/MD.0000000000023580 33655905 PMC7939229

[56] Mustafa M, Terair S, Al Ageeli E, Gohal G, Salih S. What are the Attitudes of Medical Students at Jazan University Toward Professionalism?. Adv Med Educ Pract. 2023;14:343–54. doi: 10.2147/AMEP.S399888 37057076 PMC10089272

[57] Tay KT, Ng S, Hee JM, Chia EWY, Vythilingam D, Ong YT, et al. Assessing Professionalism in Medicine - A Scoping Review of Assessment Tools from 1990 to 2018. J Med Educ Curric Dev. 2020;7:2382120520955159. doi: 10.1177/2382120520955159 33150208 PMC7580192

[58] Shaw MK, Chandratilake M, Ho M-J, Rees CE, Monrouxe LV. Female victims and female perpetrators: medical students’ narratives of gender dynamics and professionalism dilemmas. Adv Health Sci Educ Theory Pract. 2020;25(2):299–319. doi: 10.1007/s10459-019-09919-z 31541318

[59] Dertli S, Gunay U. Correlation between the occupational professionalism level and intercultural sensitivity of pediatric nurses: Case of Turkey. International Journal of Caring Sciences. 2022;15(1):19–27.

[60] Alfaris E, Irfan F, Alosaimi FD, Shaffi Ahamed S, Ponnamperuma G, Ahmed AMA, et al. Does professionalism change with different sociodemographic variables? A survey of Arab medical residents. Ann Med. 2022;54(1):2191–203. doi: 10.1080/07853890.2022.2105390 35989634 PMC9397477

[61] Wiśniewska MZ, Grudowski P. The culture of excellence and its dimensions in higher education. TQM. 2023;36(2):593–615. doi: 10.1108/tqm-11-2022-0325

[62] Guppy JH, Widlund H, Munro R, Price J. Incivility in healthcare: the impact of poor communication. BMJ Lead. 2024;8(1):83–7. doi: 10.1136/leader-2022-000717 37419661

[63] Pirzadeh A, Kamran A, Hasanzadeh M. The Relationship between Professional Identity, Performance and Attitude to Medical Errors Self-reporting among Medical Students. J Adv Med Educ Prof. 2023;11(1):61–7. doi: 10.30476/JAMP.2022.94403.1584 36685145 PMC9846100

[64] Alfandy BP. Professional identity formation of clinical medical students during and beyond the pandemic: exploring the implementation gap through personal reflection. Korean J Med Educ. 2024;36(2):223–6. doi: 10.3946/kjme.2024.298 38835314 PMC11150932

[65] Mihan A, Muldoon L, Leider H, Tehfe H, Fitzgerald M, Fournier K, et al. Social accountability in undergraduate medical education: A narrative review. Educ Health (Abingdon). 2022;35(1):3–8. doi: 10.4103/efh.efh_305_21 36367022

[66] Bhardwaj A. COVID-19 Pandemic and Physician Burnout: Ramifications for Healthcare Workforce in the United States. J Healthc Leadersh. 2022;14:91–7. doi: 10.2147/JHL.S360163 35726282 PMC9206033

[67] Bashir A, McTaggart IJ. Importance of faculty role modelling for teaching professionalism to medical students: Individual versus institutional responsibility. J Taibah Univ Med Sci. 2021;17(1):112–9. doi: 10.1016/j.jtumed.2021.06.009 35140573 PMC8802861

[68] Chien C-W, Chloe Mo SY, Chow J. Using an International Role-Modeling Pedagogy to Engage First-Year Occupational Therapy Students in Learning Professionalism. Am J Occup Ther. 2020;74(6):7406205060p1–11. doi: 10.5014/ajot.2020.039859 33275566

[69] Al Gahtani HMS, Jahrami HA, Silverman HJ. Perceptions of medical students towards the practice of professionalism at the Arabian Gulf University. BMC Med Educ. 2021;21(1):38. doi: 10.1186/s12909-020-02464-z 33419419 PMC7792125

[70] Keshmiri F, Hosseinpour A. Interprofessional professionalism as a motivating force in interprofessional collaboration. J Med Ethics Hist Med. 2022;15:8. doi: 10.18502/jmehm.v15i8.11050 37143523 PMC10151728

[71] Brenner MJ, Boothman RC, Rushton CH, Bradford CR, Hickson GB. Honesty and Transparency, Indispensable to the Clinical Mission-Part I: How Tiered Professionalism Interventions Support Teamwork and Prevent Adverse Events. Otolaryngol Clin North Am. 2022;55(1):43–61. doi: 10.1016/j.otc.2021.07.016 34823720

[72] El-Awaisi A, Yakti OH, Elboshra AM, Jasim KH, AboAlward AF, Shalfawi RW, et al. Facilitators and barriers to interprofessional collaboration among health professionals in primary healthcare centers in Qatar: a qualitative exploration using the “Gears” model. BMC Prim Care. 2024;25(1):316. doi: 10.1186/s12875-024-02537-8 39192182 PMC11348528

[73] Mun MY, Kim MY. Influence of Hospital Ethical Climate and Nursing Professionalism on Patient Safety Management Activity by Nurses. J Korean Acad Nurs Adm. 2019;25(5):458. doi: 10.11111/jkana.2019.25.5.458

[74] El Sayed R, Mohammed N, Radwan R. Is It Better to Disclose or Conceal Medical Error When Occur? An Indicative Study from Sohag Governorate Physicians. Ain Shams Journal of Forensic Medicine and Clinical Toxicology. 2021;37(2):116–27. doi: 10.21608/ajfm.2021.182704

[75] Immonen JA, Richardson SJ, Sproul Bassett AM, Garg H, Lau JD, Nguyen LM. Remediation practices for health profession students and clinicians: An integrative review. Nurse Educ Today. 2023;127:105841. doi: 10.1016/j.nedt.2023.105841 37257291

